# Comment on: Metabolic comorbidities and post-transplant outcomes in Metabolic dysfunction-Associated Steatotic Liver Disease (MASLD): a cohort study from a Brazilian tertiary center

**DOI:** 10.20945/2359-4292-2026-0023

**Published:** 2026-03-19

**Authors:** Zainab Mumtaz, Sumiaya Talpur, Hoorain Talpur

**Affiliations:** 1 Liaquat University of Medical and Health Sciences, Jamshoro, Pakistan; 2 Ibn-e-Sina University, Mirpurkhas, Pakistan

Dear Editor,

We read the paper by Caprini and cols., entitled “Metabolic comorbidities and post-transplant outcomes in Metabolic dysfunction-Associated Steatotic Liver Disease (MASLD): a cohort study from a Brazilian tertiary center” with great interest ^(1)^. The article is commendable, addressing the rising issue of MASLD, its association with increased liver transplants, and post-transplant outcomes. However, there are some methodological weaknesses which deserve a mention.

Initially, a notable methodological limitation of the study is immortal time bias in MASLD classification. MASLD was treated as a fixed exposure, but its diagnosis through imaging requires long enough survival. Early mortalities occurring in patients who could have been diagnosed with MASLD over time were automatically grouped with non-MASLD, leading to underestimated MASLD post-transplant hazards and biased comparison between MASLD and non-MASLD post-transplant survival analysis. Recent studies illustrate that observational studies are highly vulnerable to immortal time bias ^(2)^. Categorizing MASLD as a time-dependent exposure and controlling for immor-tal time bias would have strengthened the survival analysis.

Secondly, a notable limitation is failure to stratify MASLD group based on fibrosis stage. Evidence suggests that fibrosis stage, rather than steatosis alone, is a key determinant of long-term outcomes, primarily mortality, in MASLD. A recent meta-analysis demonstrates that mortality risk in MASLD rises in parallel with fibrosis stage, highlighting fibrosis stage as an important prognostic determinant ^(3)^. The lack of stratification by fibrosis stage may have obscured important prognostic differences among MASLD recipients and potentially affected the observed survival analysis.

Thirdly, the study is limited by failure to report pre-transplant diagnostic methods for MASLD, introducing misclassification bias in categorization of post-transplant recurrent and de novo MASLD. As Sharma and Arora emphasize, pre-transplant histological assessment is crucial to ensure mild or subclinical steatosis does not remain unnoticed. No histologic feature can accurately distinguish between post-transplant de novo and recurrent MASLD ^(4)^. This limitation may have caused overestimation of post-transplant de novo MASLD cases and mitigated important prognostic differences between the two groups.

Lastly, a key limitation of the article is the risk of residual confounding due to the immunosuppressive therapy administered after transplantation. Medications used for immunosuppression, such as corticosteroids, tacrolimus, and sirolimus, can lead to metabolic issues such as weight gain, dyslipidemia, insulin resistance and diabetes, all of which increase risk of MASLD. The study failed to adjust for specific type, dosage, or modifications of immunosuppressive regimens over time. This suggests that some of the outcomes may reflect the metabolic effects of these medications rather than MASLD itself. Kang and cols., highlight that different immunosuppressive regimens are associated with distinct risks of post-transplant MASLD, indicating that immunosuppressant exposure is an important confounding factor that should be considered in analyses ^(5)^.

In conclusion, the study is commendable. Further research with controlling for immortal time bias, fibrosis staging, standardized reporting of pre-transplant diagnostic methods and immunosuppressive therapy would strengthen survival analysis and clinical reliability of study.

## Data Availability

the datasets used and/or analyzed during the current study available from the corresponding author on reasonable request.
